# How should we fund end-of-life care in the US?

**DOI:** 10.1016/j.lana.2022.100359

**Published:** 2022-09-05

**Authors:** Karolos Arapakis, Eric French, John Jones, Jeremy McCauley

**Affiliations:** a Center for Retirement Research at Boston College, Boston, USA; b University of Cambridge, Cambridge, UK; c Institute for Fiscal Studies, London, UK; d Federal Reserve Bank of Richmond, Richmond, USA; e University of Bristol, Bristol, UK

**Keywords:** End-of-life care, Nursing home care, Medical spending, United States

Dying is expensive in America. Healthcare expenditures from all payors (public and private) total $80,000 in the last 12 months of life and $155,000 in the last 3 years.^1^ Although most end-of-life expenses are paid by insurers such as Medicare and Medicaid, the amount households pay out-of-pocket is hardly trivial. Furthermore, some conditions, such as dementia, are not well insured, leaving families with potentially enormous liabilities. In this viewpoint, we discuss the current funding of end-of-life care in the US. We argue that long-term care (LTC) expenses are underinsured relative to other types of late-in-life care, such as hospital spending and doctor visits. We then discuss potential reforms that would better insure families against catastrophic expenses related to LTC.

## How is end-of-life care currently funded?

**Figure 1:**
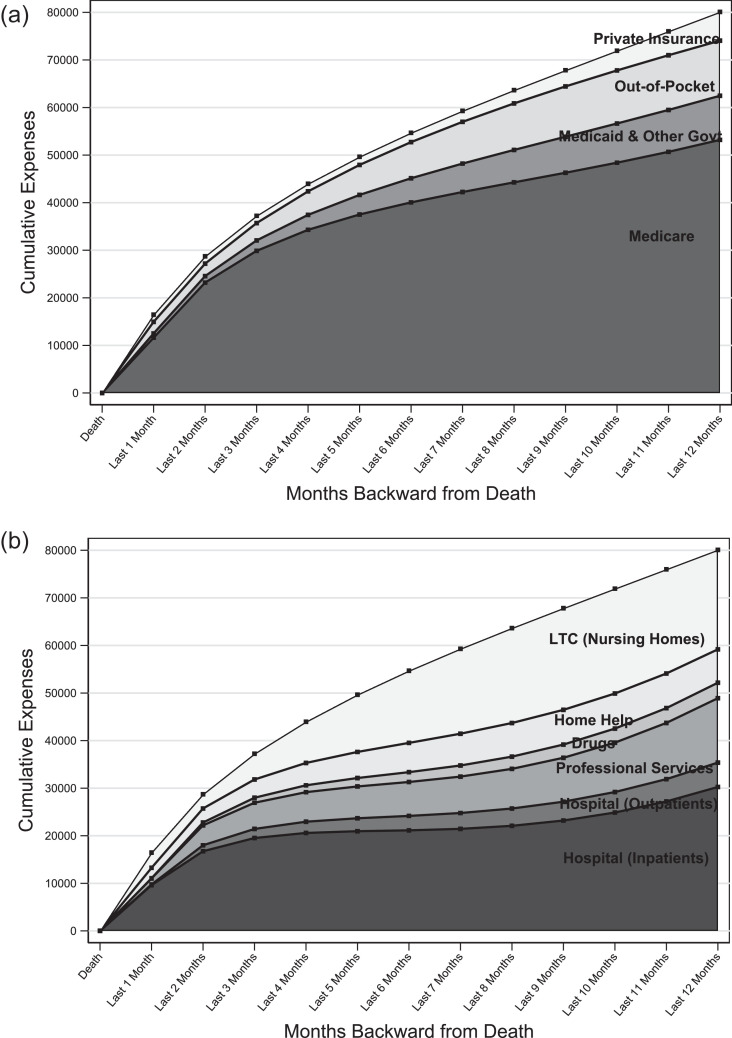
Average Healthcare Spending in the Last 12 Months of Life in the U.S., in 2014 dollars.^3^

Figure 1 shows cumulative average healthcare spending over the last year of life in the U.S. Panel A shows that of the $80,000 incurred over the last year, 66% is paid by Medicare (available to almost everyone 65 or older), 9% by Medicaid (available subject to means-testing, that is, having limited financial resources), 2% by other government programs, 8% by private insurers, and 12% ($9,500) out-of-pocket by households themselves. Given that 19% of healthcare is paid out of pocket for the over-65 population, end-of-life expenses are relatively well-insured.^2^ However, these averages mask considerable heterogeneity in both the total amount spent and the share paid out of pocket. Out-of-pocket expenses can be so high that the decedent's estate is insufficient to cover them. As debt cannot be inherited, 3% of all end-of-life charges go unpaid.

End-of-life care is relatively well-insured because much of this spending is on hospital care, which is mostly covered by Medicare. Traditional Medicare also covers hospice care for patients who have a life expectancy of less than 6 months and 80% of the cost of doctor visits. Furthermore, many people have private Medigap policies that pay for the remaining expenses or use a Medicare Advantage policy (Medicare Part C) that pays for most medical services.

Medicare coverage of LTC in the form of nursing home care is less comprehensive. Medicare pays only for skilled nursing care such as rehabilitative services, but most LTC consists of unskilled custodial care. Furthermore, Medicare pays for at most 100 days in a nursing home. Around 28% of LTC expenditures are paid out of pocket with another 8% from LTC insurance.^4^ The largest LTC payer, covering almost 30%, is Medicaid. While Medicaid rules are complicated, people in nursing homes typically qualify by having low financial resources. This includes not only the lifetime poor but also middle-class households who exhaust their wealth during a long nursing home stay. While the latter provision makes Medicaid valuable to higher-income households, it also leaves a significant part of their wealth unprotected, as such households qualify only after spending down their assets.^4^

Relative to other types of spending, LTC is more concentrated at the end of life, and a higher share is spent out of pocket.^5^ Panel B of Figure 1 shows that about one-quarter of medical spending in the final 12 months of life is for LTC, with another 9% going toward home help. If we extend the definition to cover the final three years of life, then 33% of end-of-life spending goes towards LTC.^3^ In contrast, only 6.3% of aggregate medical spending is for LTC.^4^ LTC is thus an important component of end-of-life spending in general and, given that these averages include people with no LTC needs, even more important to many households.

Because Medicare and Medicaid cover different services and operate very differently, people with different health conditions may be insured to very different degrees. Conditions requiring LTC, such as dementia, are insured relatively poorly: people who die from dementia have significantly greater out-of-pocket expenditures in the last 5 years of life ($66,000 per decedent, in 2014 dollars) than those who die of heart disease ($31,000), cancer ($38,000), or other causes ($39,000).^3^^,^^6^ The gap in the out-of-pocket burden between dementia and non-dementia decedents is especially pronounced among lower education and minority groups. This financial burden does not include the informal care provided by family and friends, which is often ignored in end-of-life expenditure analyses. The value of informal care ($89,000, based on private market rates) provided to dementia decedents is twice that of other decedents, potentially due to the higher care needs related to the disease, making the overall burden even greater.^6^

## Private long-term care insurance

If LTC expenses pose a significant financial risk, we might expect to see extensive use of LTC insurance products. In practice, however, only about 10% of older U.S. households hold private LTC insurance.^7^ Among those at the bottom of the income and wealth distribution, the share purchasing insurance is even smaller, despite higher dementia prevalence.^8^ Moreover, LTC insurance policies typically provide only partial insurance: for example, contracts usually cap both the daily payment and the number of days covered over the life of the policy.^9^ This situation is not unique to the US: the private LTC insurance market is also very small in Europe, across a variety of institutional settings.

The limited usage of private LTC insurance stands in sharp contrast to the heavy usage of Medigap policies, which provide supplemental coverage for “gaps” in Medicare's coverage but not LTC.^10^ This is rather surprising, as economic theory suggests that insurance should be most valuable when it protects against catastrophic risks, a criterion far more likely to apply to LTC insurance.

Understanding why the demand for LTC insurance is so low is essential in assessing potential alternatives to current funding schemes. The low rate of LTC insurance purchases could imply that LTC spending risk is simply not a major concern for older households.^7^ However, there are several other potential explanations. The first is that Medicaid “crowds out” private insurance. While private insurance may help households afford higher quality care, it will mostly just displace Medicaid payments because Medicaid is the “payer of last resort” and will fund care once the household runs out of assets.^11^ Second, premia for LTC insurance policies are often marked up substantially above expected claims. This is partly due to high administrative costs. It may also be partly due to those with higher LTC risk, or less access to informal care, being more likely to purchase LTC insurance. This “adverse selection’’ of purchasers can lead to higher costs or even market collapse. Research shows that although both mechanisms contribute to the low take-up of LTC insurance, Medicaid crowdout is more important for poorer individuals and adverse selection for richer individuals.^12^^,^^15^

## A role for government insurance?

Given that LTC is not well insured, there is a role for expanded government insurance. By providing insurance to all, high-risk or low, governments can create a balanced risk pool without spending money to screen out high LTC risk applicants or administer Medicaid means tests.

**Figure 2:**
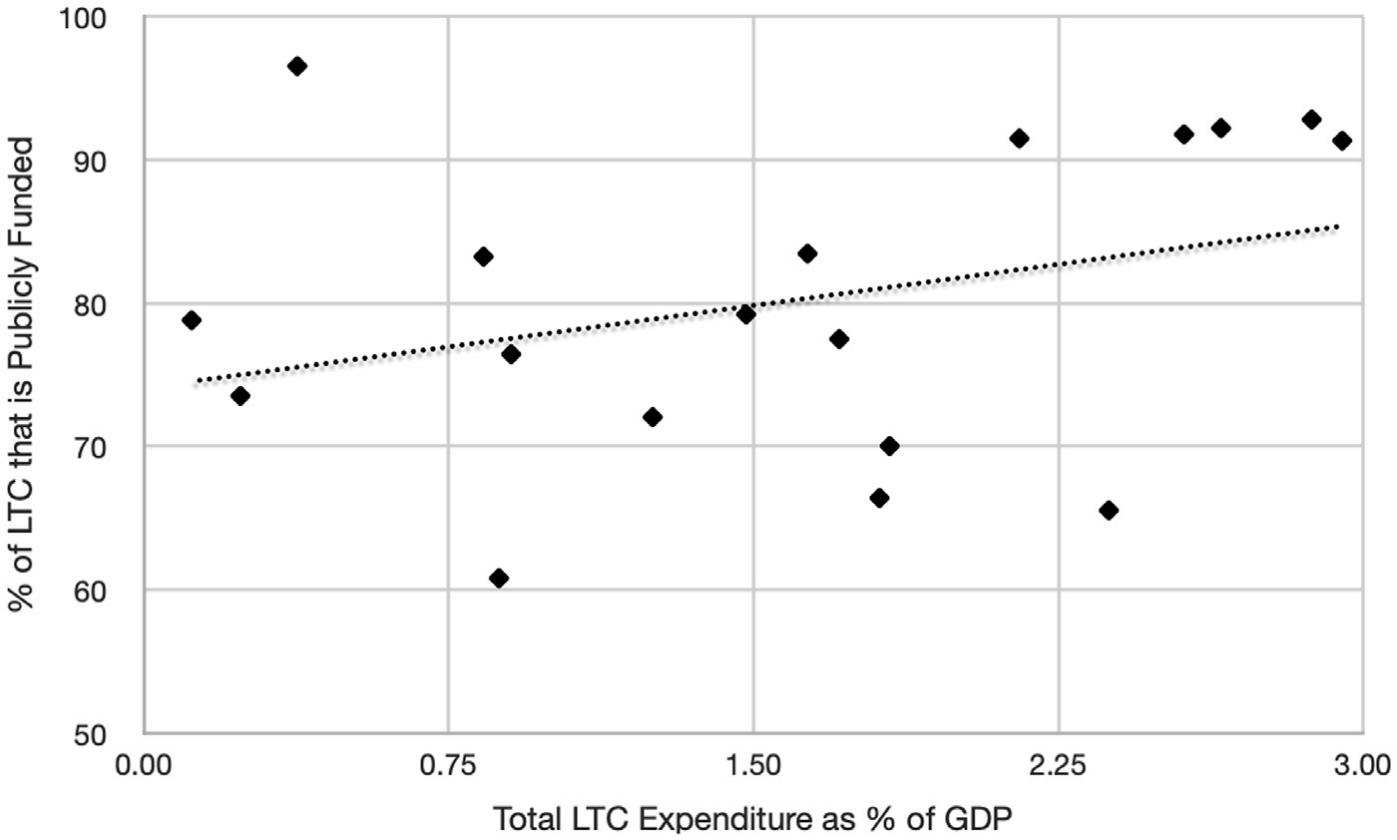
Long-Term Care Expenditures in the OECD, as Percentages of GDP, 2016.^3^

A problem for any public or private LTC insurance scheme is that it encourages families to switch from informal to formal LTC, even when formal care is of modest value, driving up overall costs—a problem known as *moral hazard*.^13^, ^14^, ^15^, ^16^ Both microeconomic evidence and cross-country comparisons lend support to these concerns. Figure 2 shows that Scandinavian countries and the Netherlands, which provide universal publicly-funded LTC, spend a very high share of GDP on LTC. In contrast, the U.K. and the U.S., which have means-tested public programs for LTC, spend a more modest share of their GDP on LTC. These differences in spending are likely due to families replacing institutional care with informal care in countries with low public LTC funding.^16^

Fortunately, cross-country comparisons also hint at some potential solutions for these moral hazard problems. For example, even with nationalized medical care, public LTC in the UK is means-tested much like Medicaid. The UK government is planning to implement some of the recommendations from the Dilnot Commission, which will put a cap on lifetime out-of-pocket LTC spending.^17^ Expenditures below the cap will (potentially) be paid out of pocket but catastrophic costs will be paid by the government. Another mechanism for reducing moral hazard would be to require families receiving LTC assistance to make co-insurance payments, as is the case in Japan. Co-insurance has been shown to reduce total expenditures.^18^

Multiple countries are experimenting with either subsidizing informal care (in particular, Germany), or giving the recipients of the care more power to choose where their allocated money is spent (e.g., to pay their informal caregiver), providing cash benefits based on the insured's health (e.g., failures in Activities in Daily Living) and allowing beneficiaries to spend the money on the care they value the most.^19^^,^^20^ This would reduce the distortion of incentives toward formal care and may be more attractive to older households, who could use their benefits to compensate informal caregivers. In fact, in the US the proposed but abandoned CLASS Act allowed for exactly this flexibility, and the currently proposed Better Care Better Jobs Act provides more opportunities for paid in-home care.

Introducing such flexibility in a public insurance program, however, will almost surely increase the number of households claiming benefits.^21^ One potential way to lower the government's obligations would be to set cash or informal care benefits below the compensation given for formal care, with the idea that beneficiaries would accept lower payments in exchange for flexibility; this is done in some countries,^14^ but the balance between encouraging informal care (and supporting informal caregivers) and limiting expenditures is a delicate one.

## Conclusion

Although end-of-life medical expenditures in the US are often large, they are generally well-insured, with most expenses covered by Medicare, Medicaid or private insurance. One important exception is LTC, where public assistance is means-tested and private insurance take-up is low, leaving middle-income, middle-wealth households vulnerable to catastrophic LTC expenses. While expanding government coverage would reduce the exposure of older households to catastrophic risk, it would also encourage households to purchase more LTC services and to replace informal care with formal. Cross-country comparisons show that controls must be put in place to avoid runaway costs. When care becomes largely free, as in the Netherlands, LTC can impose an unnecessarily large burden on society. But modest reforms that focus on catastrophic expenses or incentivize informal care can provide valuable insurance to families at a more affordable cost. Possible strategies for reducing moral hazard and limiting expenditures include lifetime spending deductibles (as in the Dilnot proposal), co-insurance, and allowing benefits to be used for informal or in-home care (as in the Better Care Better Jobs Act).

## Contributors

All four authors contributed equally to all parts of this Viewpoint.

## Declaration of interests

We declare no competing interests.
